# COVID-19 Vaccine Hesitancy in Middle-Aged and Older Adults in India: A Mixed-Methods Study

**DOI:** 10.7759/cureus.30362

**Published:** 2022-10-16

**Authors:** Nidhi Sanghavi, Elena Neiterman

**Affiliations:** 1 School of Public Health Sciences, University of Waterloo, Waterloo, CAN

**Keywords:** covid-19 vaccine india, pandemic, covid-19, mixed-methods study, fear of side-effects, government distrust, misinformation, indian health care, vaccine hesitancy

## Abstract

Introduction: Vaccine hesitancy is a significant threat to public health efforts to stop the negative impacts of the COVID-19 pandemic. In India, it is critical to attain high vaccination rates to prevent overload in the healthcare system. Older adults play a central role in families’ decision-making, but there is a lack of research on middle-aged and older adults' vaccine perceptions in India in general, and about their concerns about COVID-19 vaccinations.

Research question: This study aimed to explore which factors affect COVID-19 vaccine hesitancy in middle-aged and older adults in India and what factors can reduce their vaccine hesitancy and increase its uptake.

Materials and methods: A mixed-method sequential design was employed to conduct the study. Convenience sampling was used to recruit participants by sending an online invitation. For phase one of the study, a quantitative survey with 34 questions was distributed through WhatsApp. For phase two of the study, qualitative one-on-one interviews were conducted with those participants who completed the survey and agreed to participate in this next phase.

Results: In total, 65 individuals responded to the online survey and 10 participated in semi-structured interviews. The participants were residing in India and their age range was from 40 to 89 years. Analysis of the data identified that although the majority of participants supported the vaccine, the main reasons for vaccine hesitancy included uncertainty about the effectiveness of the vaccine, fear of side effects, unclear and insufficient information about the vaccines and altered risk perception. This study also showed that those who felt that the consequences of COVID-19 were mild were also more likely to be vaccine-hesitant.

Conclusion: While the results of the study showed that most of the participants supported the COVID-19 vaccines, they expressed uncertainty regarding their effectiveness. The safety and effectiveness of the vaccines were found to be prime contributing factors to vaccine hesitancy in this sample. The findings from this pilot study can be used to develop a larger, more comprehensive study on vaccine hesitancy among middle-aged and older adults in India, which would provide more insights into strategies that can be employed to promote vaccinations.

## Introduction

In December 2019, the world saw the first case of COVID-19 in Wuhan, a city of Hubei province, China, and the WHO declared a pandemic on March 11, 2020 [1]. The symptoms of this novel and rapidly spreading disease were largely unknown but were shown to impact physical, mental, and cognitive health [2]. The pandemic had caused enormous disruption to human life and the world economy, presenting an extraordinary challenge to global health [3]. With the rise in the number of cases and COVID-19-related deaths, vaccine development was accelerated, and several pharmaceutical companies began large-scale clinical trials to test new vaccines, including those that were in the early stages of development [3]. There was a rapid development of vaccines to tackle the SARS-CoV-2 virus, particularly by BioTech, Moderna and Johnson & Johnson [3]. In India, three vaccines, Covishield, developed by the Serum Institute of India, Covaxin, developed by Bharat Biotech, and Sputnik V, developed by the Gameleya Research Institute of Epidemiology and Microbiology, also gained approval from the Central Drugs Standard Control Organisation (CDSCO) [4].

The COVID-19 outbreak in India began in Thrissur, Kerala after a few students returned from Wuhan, China [5]. The first wave of the COVID-19 pandemic in India began in March 2020 with thousands of daily infections, but by February 2021, the curve of COVID-19 cases had flattened. However, the spiralling cases during the second wave of the pandemic in March 2021 led to devastating conditions of an overworked health care system, a limited supply of hospital beds, oxygen, medications, ventilators, and rapidly increasing mortality rates [6]. India saw the worst of the pandemic during this period as a result of the high infection rate of the new mutants of the SARS-CoV-2 virus, especially the double-mutant strain of the Alpha (B.1.1.7) and Delta variant (B.1.617) [7]. The phenomenal speed of infection and the rise in reproduction number (R0) during the second wave of the pandemic can be attributed to the confluence of numerous factors: lack of preparedness of the health care system, non-compliance with social-distancing norms, increased testing, political elections, poorly implemented precautions during festivals and weddings, sporting events, and large-scale religious gatherings like the Haridwar Kumbh Mela and the Tablighi Jamaat [6,8,9]. The Indian health care system cracked under pressure and was unable to keep up with the volume of COVID-19 cases [10]. During this time, India was recording more than 400,000 daily infections and the highest number of deaths over 4,000 in a single day [10]. This was India’s worst battle against the virus, as graveyards ran out of space, round-the-clock mass cremations were conducted, and hospitals turned away patients due to the lack of beds and medical supplies [10].

As per the Indian Constitution, health care is the responsibility of the individual states and not the central government [11]. The national health care organization, the Union Ministry of Health & Family Welfare (MoHFW), is the organization responsible for any national-level health care programs and health policy and planning [11]. Despite the continued efforts of the state governments to control the spread of COVID-19, their health care system was crippled under pressure and required leadership from the central government [9]. In this situation, the constitutional limits were crossed, and the central government stepped in to take charge of the situation [9]. Owing to India’s population density, differences in health literacy and administrative barriers, vaccinating the entire population was going to be a massive undertaking [11]. The vaccination drive in India began on January 16, 2020 [4]. The central government also launched a mobile application called CoWin (Covid Vaccine Intelligence Network) for self-registration of vaccination slots, along with monitoring and surveillance of the number of doses administered [4].

In India, a certain level of vaccine hesitancy was expected with the novel coronavirus disease [4]. Several challenges to achieve high vaccination rates had already sprung up and undermined efforts to control the pandemic [4]. A limited number of vaccine slots on the CoWin app, vaccine shortage and cost of vaccines had posed challenges in the early phases of the vaccine drive [4]. Initially, they were not offered free of charge, and the price of the vaccine steadily increased and varied from one hospital to another [12]. Furthermore, barriers to registering on the government website, which was initially available only in English, intensified inequalities, deepening the technological divide in the country [13]. However, the government has modified the program by waiving pre-registration on the portal and making vaccines free for all [4]. Affordability, availability, and access to vaccines due to the severe demand and supply mismatch were major concerns [4]. Despite the extensive measures to provide information about the precautions and vaccination plans through telecommunication platforms, the rampant spread of COVID-19 misinformation has been posing major threats to vaccine uptake [14]. For example, myths about vaccines causing infertility and disrupting the menstrual cycle, consuming alcohol to treat COVID-19, previous Bacillus Calmette-Guérin (BCG) immunization as an effective measure to prevent COVID-19 infection and a previous infection of malaria making a person immune to COVID-19 have been leading to vaccine hesitancy, especially in rural areas [4]. Misleading information spread in Hindu and Muslim communities relating to the vaccines containing pork and aborted fetal tissue also proved detrimental to the vaccine drive [14].

The existing literature does a fair job of assessing the concerns about the COVID-19 vaccine, including in India [15,16], but there is a paucity of research on the perceptions regarding COVID-19 vaccinations among middle-aged and older adults in India. A narrative review by Troiano et al. looked at vaccine hesitancy in students and the general population in India [17]. Additionally, a study by Umakanthan et al. also documented the results of a national survey, demonstrating the importance of vaccination coverage within the country [18]. However, none of the studies focused on middle-aged and older adults in India. In many Indian families, the elders in the family are the primary decision-makers that influence overall behaviours and practices [19]. Due to these cultural beliefs and the importance of elders in the family, it is fundamental to understand the vaccination perceptions of this age group since they are largely responsible for health-related decision-making in the family. The goal of this paper is to address this gap and examine what are the major factors that affect COVID-19 vaccine hesitancy in middle-aged and older adults in India. Drawing on a small, convenience sample of middle age and older adults residing in India, this paper aims to offer initial insights about attitudes towards vaccinations and COVID-19 vaccines in this population group.

## Materials and methods

This exploratory pilot study utilized a sequential mixed-methods design where online quantitative surveys were followed by qualitative individual interviews. After receiving ethics clearance from (blinded for peer review), an invitation for an online, anonymous survey was distributed to a small sample of people utilizing convenience sampling. WhatsApp social media platform was used to recruit participants. The recruitment message contained a link to the online survey.

Data collection and analysis

*Quantitative Online Survey* 

The survey, containing 34 items on vaccine hesitancy was distributed in September 2021, when the Delta strain initiated the second wave of the pandemic in India. Survey development was based on a validated scale from a study by Wong et al. [20] and the questions were modified to be compatible with the context of the COVID-19 pandemic in India. The questionnaire consisted of several sections that covered demographics, vaccine acceptability, perceived severity, susceptibility, benefits, barriers, level of trust in the government, and individual behaviour. To assess their level of agreement, concern, severity, and likelihood with respect to COVID-19, the variables were measured on a 5-point Likert scale. For example, to assess the level of concern, participants were asked “How concerned are you about the following?” with the following sub-questions: (1) side effects; (2) ingredients; (3) safety; and (4) effectiveness of the vaccines. Participants had the following response options: 1=Not at all concerned; 2=slightly concerned; 3=Somewhat concerned; 4=Moderately concerned; and 5=Extremely concerned. Other socio-demographic predictor variables like (1) age; (2) gender; (3) perceived health status; (4) primary decision-maker; (5) family composition; (6) annual family income; (7) ethnic background; (8) level of education; (9) marital status; and (10) geographic location had response options clustered into categorical groups.

The survey was housed on the Qualtrics platform and opened up with information about the study. Participants consented to the survey by clicking the "I agree" button to indicate their willingness to participate. At the end of the survey, the participants were asked if they would be willing to be contacted for the one-on-one interview, and those who consented were prompted to provide their email addresses for future contact. Through the anonymous online survey, the quantitative data collected on Qualtrics was exported to Excel where descriptive analysis and summary statistics were used for data analysis. Descriptive statistics were used to highlight any relationships and patterns between the data obtained using histograms and pie charts [21]. The data were organized and coded by converting the responses into a numeric format to extract themes and correlations.

Qualitative One-on-One Interview

For Phase two of the study, 10 one-on-one interviews were conducted with individuals who agreed to be contacted for a follow-up interview. These interviews were conducted during the third wave of the pandemic in February and March 2022 when the Omicron strain was prominent. Questions were open-ended and included short demographic probes about age, gender, family composition, and decision-making powers, as well as more in-depth questions about participants’ overall response to the COVID-19 pandemic, as well as about their perceptions about COVID-19 vaccines’ safety and efficacy. Post transcription and removal of identifiers from the open-ended interviews, a thematic analysis framework was used to analyze qualitative or textual data to generate codes and identify themes [22]. Braun and Clarke’s six-step repetitious method was utilized for coding and generating themes to interpret and report the findings [22].

This involved: (1) familiarization with the data, (2) initial coding from related comments, (3) generating preliminary thematic groups, (4) reviewing themes, (5) defining themes, and (6) interpretation and reporting. The participants’ profile is summarized in Table 1. 

**Table 1:** Socio-demographic profile of interview participants

Variables	Number (%)
Age	
40-50	3 (30%)
51-60	4 (40%)
61-70	1 (10%)
71-80	1 (10%)
81-90	1 (10%)
Gender	
Male	4 (40%)
Female	6 (60%)

Final Data Analysis

**Figure 1:**
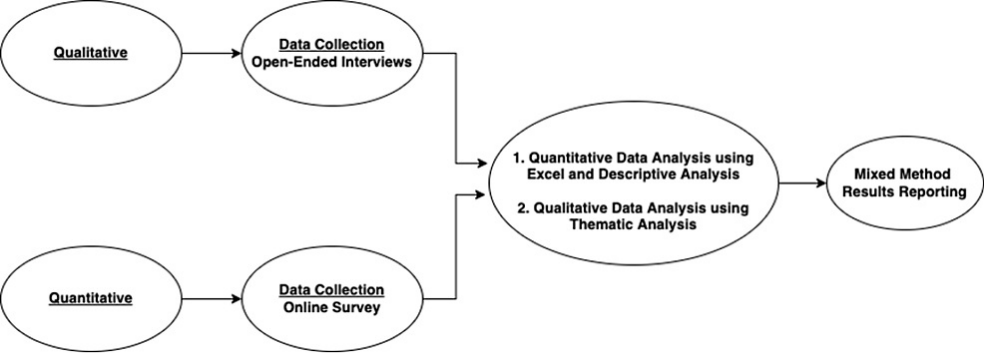
Steps involved in data collection and data analysis

Once quantitative and qualitative data analyses were completed, a convergent parallel design was utilized for the final interpretation. This concurrent methodology enabled the independent collection of diverse yet complementary data during a similar timeframe and helped merge the results to obtain a comprehensive evaluation [23]. The themes and patterns identified during quantitative and qualitative data analysis were used for a side-by-side comparison jointly displaying both forms of data [23]. For this purpose, tables and figures were used to summarize the findings and concisely present them by the major themes identified. Figure 1 demonstrates the steps involved in the data collection and analysis process.

Respondent Profile

A total of 65 people participated in the survey. Six responses were discarded because the surveys were incomplete, or the age criteria were not met. Thus, the final number of responses for the survey was 59. Table 2 summarizes the demographic profile of the survey respondents. The average age of the participants was 53 years. Among the participants, 61% (n=36) self-identified as women and 39% (n=23) as men. In this participant group, 67% (n=40) of them held an undergraduate degree, most of the respondents were married (90%, n=53) and lived with four-six people in their households (66%, n=39).

**Table 2:** Socio-demographic profile of survey participants

Variables	Number (%)
Age	
40-50	29 (49.15%)
51-60	21 (35.59%)
61-70	6 (10.16%)
71-80	2 (3.38%)
81-90	1 (1.69%)
Family Composition	
1-3 people	11 (18.64%)
4-6 people	39 (66.10%)
7-9 people	7 911.86%)
10+ people	2 (3.38%)
Family Income	
5 Lakhs or less	3 (5.08%)
Between 5 and 10 Lakhs	11 (18.64%)
Between 10 and 20 Lakhs	13 (22.03%)
Between 20 and 40 Lakhs	17 (28.81%)
Above 40 Lakhs	12 (20.33%)
Other	3 (8.47%)
Level of Education	
Grade 10	1 (1.69%)
Grade 12	2 (3.38%)
Undergraduate degree	18 (30.50%)
Post-graduate degree	22 (37.28%)
Doctorate/Ph.D.	1 (1.69%)
Certificate Degree	9 (15.25%)
Diploma Degree	2 (3.38%)
No formal education	1 91.69%)
Other	3 (5.08%)
Marital Status	
Married	53 (89.83%)
Never married	5 (8.47%)
Prefer not to answer	1 (1.69%)
Perceived Health Status	
Somewhat bad	4 (6.77%)
Neither good nor bad	13 (22.03%)
Somewhat good	29 (49.15%)
Extremely good	13 (22.03%)
Decision Making Capacity	
I am the primary decision-maker in my family	16 (27.11%)
My partner is the primary decision-maker in the family	10 (16.94%)
My partner and I are the primary decision makers in the family	24 (40.67%)
My parents/parents-in-laws are the primary decision-makers in the family	7 (11.86%)
No one person is the primary-decision maker in my family	2 (3.38%)

**Figure 2:**
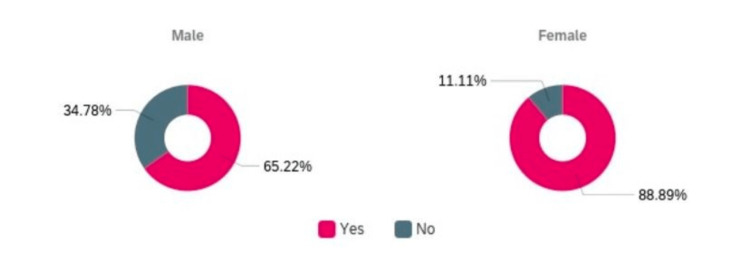
Proportion of men and women who received and did not receive a vaccine for COVID-19

At the time they completed the survey, 80% (n=47) of the participants had received a vaccine for COVID-19 and most of them (73%, n=43) had received both doses. Of the people who got the vaccine, 24% (n=14) waited for others to get the vaccine before scheduling a shot. Results from the quantitative survey also showed that women (89%, n=32) were more likely to receive the COVID-19 vaccines when compared to men (65%, n=15) as seen in Figure 2.

## Results

After analyzing the quantitative and qualitative data collected, three key themes were identified during the analysis: (1) Perceptions about COVID-19 disease and COVID-19 vaccine in India; (2) Safety, Efficacy, and Availability of COVID-19 Vaccines; and (3) Public Health Promotion and Education. These themes are presented below and are accompanied by the survey data results. 

Perceptions about COVID-19 disease and COVID-19 vaccine in India

As seen in Table 3, more than half of the respondents (54%, n=32) ‘strongly’ and ‘somewhat’ agreed that their friends and family were at risk of getting COVID-19, and most believed that they were at risk of contracting COVID-19.

**Table 3:** Level of agreement about risk of COVID-19

To what extent do you agree or disagree with the following?	Strongly disagree	Somewhat disagree	Neither agree nor disagree	Somewhat agree	Strongly agree
You are at risk of COVID-19 infection	11 (18.64%)	10 (16.94%)	10 (16.94%)	21 (35.59%)	7 (11.86%)
You are immune to COVID-19	18 (30.50%)	12 (20.33%)	16 (27.11%)	7 (11.86%)	6 (10.16%)
Your friends and family are at risk of getting COVID-19	8 (13.55%)	8 (13.55%)	11 (18.64%)	23 (38.98%)	9 (15.25%)

As shown in Table 4, more than half of the participants thought that the government was doing a good job at controlling the COVID-19 pandemic and only 20% felt it did not do a good job. Furthermore, 49% of the participants also reported that they had trust in the ability of the Indian health care system to deliver timely care during the pandemic.

**Table 4:** Questions regarding level of trust in the government

	Yes	Maybe	No
Do you think the government is doing a good job in controlling the pandemic?	35 (59.32%)	12 (20.33%)	12 (20.33%)
Do you have trust in the ability of the Indian health care system to deliver timely care during the COVID-19 pandemic?	29 (49.15%)	15 (25.42%)	15 (25.42%)

The analysis of the data derived from the interviews supported the survey results. About half of the participants displayed high levels of trust in the Central government and the way the government has been handling the pandemic. For example, Participant 2, a 77-year-old-woman, noted:

“It (government) is giving us two doses as per its duty, and I fully trust the Indian government and appreciate Narendra Modi for the commendable success of the vaccine drive and for the speedy manufacturing of the vaccines.” (Participant #2)

This participant took pride in the nation’s success with the COVID-19 vaccine drive and urged other people to follow the guidelines, displaying support for the government. Similarly, Participant 6, a 54-year-old-man, also showed support for the government by expressing his trust in the national COVID-19 vaccine drive when he said:

“See, the new variants are what, nobody's got control on them. Our policies are good for vaccines. I don’t know if it is effective but it is the need of the hour. So I wouldn't blame the government for anything like future infections. The government has laid out rules and is helping vaccines to be supportive, but there's no guarantee in life, the government or anybody cannot give you a guarantee.” (Participant #6)

Evidently, while Participant 6, like many others, was unsure about the impact of new variants, he still perceived the government policies to be *“good”* for fighting COVID-19.

However, not all participants trusted the government or saw COVID-19 as a real threat. Some, like Participant 8, a 45-year-old woman, had a different perspective: 

“I have not been scared at all. We have to live this way, there is nothing to fear. Its.. it's like the flu, it's like a common cold. It comes and goes away. There is no reason to be scared. My daughter works in a lab, but I'm not worried because we all are very healthy and we eat very good food, so there is no reason to be scared during the pandemic. I think the best way to avoid COVID-19 is by taking homeopathic medicines. I'm drinking warm Haldi milk and chyawanprash every single day. You see here, these antiseptics will cure everything, and they will make your body stronger. Just rely on home remedies and focus on eating well.” (Participant #8)

Comparing COVID-19 to a “common cold”, Participant 8 normalized the disease, presenting it as insignificant and not particularly scary. This can be attributed to the period in which the interviews were conducted where India saw the emergence of the highly transmissible but less deadly Omicron variant which led to fewer hospitalizations despite the rising number of infections. The participant also referred to the use of alternative medicine to prevent the onset of COVID, a conviction that made her not fearful of contracting COVID.

When the concerns about COVID were minimized, it also affected individuals’ decisions to get a vaccine. For instance, Participant 9, a 45-year-old man, discussed the shift towards working from home and having less contact with people as a primary reason for not getting the vaccine. He said:

“I really don’t think I need it because I have been working from home since the start of the pandemic. I haven't really been going out, so I cannot get infected. We get our groceries online. We get food delivered. We get clothes online. We don't attend weddings or any functions. Honestly, we have been going out very less frequently now. I meet very few people now. I just don't see the reason why I should get vaccinated because I am staying indoors all the time and the world is moving in that direction.” (Participant #9)

According to this participant, working from home reduced his exposure to the virus and hence reduced his risk of getting infected and the need to get vaccinated. Additionally, explaining his position about why the perception of the pandemic has changed, Participant 7, a 70-year-old-man, said:

“I think the reason is that now if anybody is falling sick, they are calling it COVID. If anybody is dying, they are calling it COVID. So there is no more fear any more I feel. Nobody cares about protocol anymore. I do not see anybody wearing masks. Basically, there is no trust in the government and what they are trying to do. I’m extremely healthy right now, and if I take the vaccine it can cause complications, so, I would like to avoid it. The government is trying to promote the vaccine, but infections are rising by the day. So what is the point? There is no justification for this. First, they say one shot, then they say another shot, and now we have to take a booster shot. I do not want to get into this cycle.” (Participant #7)

According to Participant 7, his lack of fear of COVID stems, at least in part, from his distrust of the government and the lack of consistency in the information about COVID-19. The changing instructions on vaccinations and the surge in the number of reinfections with the Omicron variant have also made Participant 7 raise concerns about the effectiveness of the COVID-19 vaccine. Like participant 8, he attributes his low susceptibility to the virus to his *“*extremely healthy*”* lifestyle. 

Close to half of the respondents ‘strongly’ and ‘somewhat’ disagreed that the COVID-19 vaccine will protect them from getting and spreading the infection as seen in Table 5 below. However, a large group i.e., 80% (n=47) also ‘strongly’ and ‘somewhat’ believed that taking the vaccine can decrease the severity of illness and the chances of complications during a COVID-19 infection. Additionally, a majority of the participants also ‘strongly’ and ‘somewhat’ agreed that the COVID-19 vaccine will provide their family members with protection against the virus.

**Table 5:** Perception of COVID-19 vaccines

To what extent do you agree or disagree with the following?	Strongly disagree	Somewhat disagree	Neither agree nor disagree	Somewhat agree	Strongly agree
COVID-19 vaccines keep you from getting and spreading the virus	17 (28.81%)	11 (18.64%)	7 (11.86%)	13 (22.03%)	11 (18.64%)
Getting the vaccine will prevent future COVID-19 infection	17 (28.81%)	7 (11.86%)	7 (11.86%)	19 (32.03%)	9 (15.25%)
COVID-19 vaccine can help me protect my family members from the disease	10 (16.94%)	10 (16.94%)	4 (6.77%)	20 (33.89%)	15 (25.42%)
COVID-19 vaccine can decrease the severity and the chance of having complications	6 (10.16%)	3 (5.08%)	3 (5.08%)	21 (35.59%)	26 (44.06%)

In the interviews, some participants expressed their belief that vaccines help to reduce the severity of COVID-19 symptoms, suggesting that with vaccines, *"people are having milder infections and are not getting hospitalized as seen in the second wave” *(Participant #6).

While the survey results suggested that participants considered themselves and their families at risk for COVID-19 infection, results from the interviews showed complexity in the perceptions regarding the disease. The low levels of perceived risk and a decline in trust in the government seemed to be important drivers for lower acceptance of the COVID-19 vaccines. The shift to working from home, especially during lockdowns also led to dismissing the pandemic as *“mild”*, which, in turn, altered the perceived risk of getting the disease.

Safety, efficacy, and availability of COVID-19 vaccines

In the survey, participants were also asked whether they thought that getting the COVID-19 vaccine was the best way to avoid complications post-infection and as seen in Table 6, the majority of the participants agreed with this statement, indicating their trust in the COVID-19 vaccines to minimize hospitalizations and prevent future complications.

**Table 6:** Level of agreement about post-COVID-19 infection complications

To what extent do you agree or disagree with the following?	Strongly disagree	Somewhat disagree	Neither agree nor disagree	Somewhat agree	Strongly agree
The best way to avoid the complications of COVID-19 is by getting the vaccine	13 (22.03%)	4 (6.78%)	6 (10.17%)	7 (11.86%)	29 (49.15%)

The interviews, however, showed the nuances in the level of trust in the COVID-19 vaccines. While most participants believed that the vaccines were effective in protecting against severe illness, they also pointed out that the COVID-19 vaccine did not offer complete protection against infection and reinfection with newer variants of the virus. Participants were also concerned about transmitting the virus to family and friends even after getting two shots of the vaccine.

Several participants also raised concerns about COVID-19 vaccines’ effectiveness referring to some of the (mis)information on their ingredients, delivery, and safety as seen in the quotes below. Participant 7, a 70-year-old man, for instance, was questioning the effectiveness of the vaccine and was also concerned about the vaccine changing fertility patterns and causing more harm than good. He said:

“A lot of people say that there are several side effects of the vaccine, some people say that you cannot have a child if you get the vaccine… like you get infertile. Some people say that you will get a heart attack if you take the vaccine. Some people say that there will be blood clots with the vaccine and you could even die so this should be a personal choice and not forced. I don’t want it.” (Participant #7)

His fear of serious adverse side effects led to the downright rejection of the COVID-19 vaccine. An important event that got a lot of media attention was the death of a well-known Tamil actor Vivekh, just two days after taking the COVID-19 vaccine during the second wave of the pandemic. Participant 5, a 50-year-old-woman highlighted this important event, noting that “many people were afraid to take the vaccine after this and thought that it had to do with the government vaccines.”

After Vivekh’s death, the increased fear of death among the people led to a change in public attitudes regarding vaccines. This was reiterated by Participant 6 who discussed how rumors and misinformation around the Indian COVID-19 vaccines affected people’s intent to get vaccinated. He said:

“There are rumours and the word is floating around that covishield and Covaxin vaccines will give you a lot of side effects. And the rumours about the vaccine being not correct or not good or being harmful and stuff like that. So that plays havoc in the minds of people, people who are gullible or people who don't have their own mindset. It really, you know, gives them a lot of trauma, and that they do it for their own political or political or their personal gains. So, that is rampant here.” (Participant #6)

Displaying concern around misinformation, he suggested that false information for political or personal gains affected vaccine uptake. Similarly, this issue regarding the lack of transparency was also seen in Participant 10, a 51-year-old-woman who said:

“Yeah, I don't trust the government. I've heard cases where they are substituting water, substituting the vaccine with water, or even giving saline solution instead of the vaccine (in) some places. I've heard that they are just injecting air. I feel like the government does not give a lot of attention to villages in small towns. It is only focused on big cities, so I don't trust the government very much.” (Participant #10)

Calling attention to the vulnerabilities of the rural population, she condemned the government’s response to COVID-19 vaccinations, highlighting the issues faced by the neglected rural areas when compared to larger, metropolitan cities. This was also intensified by the inconsistencies in the government’s pricing structure for COVID-19 vaccines. Participant 3, a 54-year-old-women said:

“Some places they were charging Rs. 500, Rs. 600 and some places they were charging Rs. 1500. Due to this, some people were substituting it with water for money. They were incentivizing people to pay more to get the vaccine faster and can save their families. Now it is free so it is proper. There is no reason for the government to give fake vaccines. Trust has increased. Free vaccination has helped everyone trust the government.” (Participant #3)

While most participants believed in vaccines, some felt that they were not effective by suggesting that: “Today's situation if we see, we are not happy about vaccination that even after taking two doses, people are getting infected for the second and third time.”* *(Participant #5), demonstrating uncertainty in the effectiveness of the vaccines. This was confirmed by the survey results where the majority of the participants were concerned about the effectiveness and the safety of the vaccine, although only 12% (n=7) expressed ‘extreme’ concern about the ingredients of the vaccine.

To further understand the challenges faced with COVID-19 vaccinations, the survey asked questions regarding individuals’ experiences with the Co-WIN application to book slots for vaccine shots, and Table 7 summarizes the results. Majority of the participants ‘strongly’ and ‘somewhat’ agreed that it was difficult to book an appointment through this app whereas 39% (n=23) of the participants ‘strongly’ and ‘somewhat’ disagreed. The struggle to find open vaccination slots and the digital divide left people with less technical know-how to wait much longer.

**Table 7:** Level of agreement about vaccine drive

To what extent do you agree or disagree with the following?	Strongly disagree	Somewhat disagree	Neither agree nor disagree	Somewhat agree	Strongly agree
It is difficult to book a vaccination appointment through the Co-WIN app	15 (25.42%)	8 (13.55%)	4 (6.77%)	11 (18.64%)	21 (35.59%)

Several other concerns were also raised around the COVID-19 vaccines during the surveys. Table 8 displays the responses of the participants on a Likert scale where the 10 statements captured their concerns surrounding COVID-19 vaccines. Overall, the primary barriers that came up were the chances of reinfection, as well as unclear and insufficient information regarding the vaccines. A majority of the participants ‘strongly’ agreed that they needed the vaccine despite previous COVID-19 infection and did not have religious reasons to question the vaccine. Fear of needles was also not found to be a major reason for concern for most of the participants.

**Table 8:** Level of concern regarding the COVID-19 vaccine

From the reasons listed below, how concerned are you about the following?	Strongly disagree	Somewhat disagree	Neither agree nor disagree	Somewhat agree	Strongly agree
I do not have enough information about the vaccine	14 (26.92%)	11 (21.15%)	6 (11.54%)	18 (34.62%)	3 (5.77%)
I am unclear of the available information about the vaccine	12 (23.08%)	11 (21.15%)	7 (13.46%)	18 (34.62%)	4 (7.69%)
I am worried that even after getting the vaccine, I can be infected with COVID-19	6 (11.54%)	5 (9.62%)	12 (23.08%)	16 (30.77%)	13 (25%)
I am anxious about vaccination in general	11 (21.15%)	8 (15.38%)	14 (26.92%)	11 (21.15%)	8 (15.38%)
Religious reasons make me question the vaccine	34 (65.38%)	5 (9.62%)	7 (13.46%)	3 (5.77%)	3 (5.77%)
I believe more in traditional home remedies	22 (42.31%)	6 (11.54%)	10 (19.23%)	8 (15.38%)	6 (11.54%)
I do not like needles	30 (57.69%)	7 (13.46%)	7 (13.46%)	3 (5.77%)	5 (9.62%)
I have no concerns about the COVID-19 vaccine	22 942.31%)	9 (17.31%)	11 (21.15%)	3 (5.77%)	7 (13.46%)

As seen in Table 9, an additional concern was related to the lack of COVID-19 information. However, 35.59% (n=21) of the participants reported that they were ‘not at all concerned’ about vaccine availability. The participants who reported their concern about the lack of COVID-19 information relied on social media (WhatsApp, Facebook, Instagram, Twitter, Snapchat, etc.) and their social circle (friends, family, neighbours, etc) as their most common source of information about the pandemic. Additionally, only 22.03% (n=13) reported their reliance on public health websites (World Health Organisation, Centre for Disease Control, Ministry of Health and Family Welfare, National Health Portal of India, etc.) for their information.

**Table 9:** Concerns about COVID-19 vaccines

How concerned are you about the following?	Not at all concerned	Slightly concerned	Somewhat concerned	Moderately concerned	Extremely concerned
Vaccine availability	21 (35.59%)	10 (16.94%)	7 (11.86%)	11 (18.64%)	10 (16.94%)
Lack of COVID -19 information	11 (18.64%)	9 (15.25%)	16 (27.11%)	19 (32.20%)	4 (6.77%)
Protection against new variants of the virus	0 (0.00%)	10 (16.94%)	10 (16.94%)	8 (13.55%)	31 (52.54%)

Survey results suggested that the majority of respondents displayed trust in the government to control the COVID-19 pandemic, deliver timely care during the pandemic and seemed to be supportive of their measures to control the virulent spread. However, participants reported challenges with the CoWIN application to book vaccine slots and a lack of COVID-19 information. Overall, the comments from the interviews seemed to indicate that the participants had several concerns with the government and the health care system. Concerns regarding reinfection, safety, and efficacy of the vaccines were prominent. However, the shift from charging vaccines to making them free nationwide seemed to have a positive impression on the participants in this study.

Public health promotion and education

When asked about the strategies to further promote the vaccine, interview participants offered several approaches to strengthen the current vaccine drive. Participant 6, with a focus on using media to spread the message, suggested increasing health promotion activities to improve awareness of COVID-19 vaccination. He said:

“I was saying you've got to educate people, you've got to instil confidence in them that this (vaccine) is not a bad thing. This is not going to harm them in any way. You do it through by way of advertisements or by way of word of mouth. You know, educating through mass media, through papers, through newspapers, through electronic media, through the internet or whichever way, but the message has to be reached to the people that this is good. And that maybe somebody would be doing but all this, you know, maybe ask a Bollywood person or Hollywood personalities to post on Instagram? If they get into this mode of promoting the vaccine? It should definitely help.” (Participant #6)

Focusing on further publicizing the vaccine’s safety and benefits as a public health intervention is especially important for educating those who might be living in rural and remote areas, as was noted by Participant 4, who said:

“Mouth to mouth..mobile.WhatsApp forwards. In villages.. education is important. If there is a gathering of people, you should let everyone know that vaccine is important. Or we can even do door-to-door as we did during polio. But that is very laborious. Door-to-door could be a potential strategy. When they go, they can educate the people at their homes and give the vaccine there itself.”(Participant #4)

Keeping in mind the enormous size of the Indian population, Participant 4 suggested that medical teams go door-to-door, especially in rural areas, due to the technological divide, to ensure that everyone has received the COVID-19 vaccine. Moreover, to focus on the use of vernacular language for health education, Participant 3 said:

“Education is the most important. Telling them about the results and showing them statistics will help people understand that they are good. Telling people in the local language is also important so they trust the information more. The stats will show them that people are not dying with the shot and improve immunity.” (Participant #3)

Region-specific transparent messaging in rural areas could help motivate people to get the COVID-19 vaccine suggesting that if paid close attention to the needs of rural India, vaccine messaging could be more effective and impactful.

Similarly, with a focus on education and building awareness for COVID-19 vaccines, Participant 9 highlighted the importance of targeting educational campaigns for people living in economically weaker areas, specifically, the largest slum in Asia, Dharavi. He stresses the importance of educating this traditionally excluded group to improve health literacy when he said:

“If you look at Dharavi in Mumbai, the largest slum, there is so much superstition and ignorance in those areas. It's such a big slum area and there has to be more awareness, at least for the people who are not educated. Many people think that the vaccine kills people. I do not think it kills people, but I think they are very scared of the side effects. I feel like there's a lot of ignorance I do not think that vaccines will 1000% protect you from the disease but if people do want to take it then they should look at the pros and cons.”(Participant #9)

He also brought up an important issue about daily-wage workers and how they are left out of the vaccination programme due to insufficient support available to help them deal with any potential side effects. He said:

"Why it should be mandatory? Also, you have to think about slums and poor people, and daily wage workers. There are so many here in India. You cannot make it mandatory because if they do fall sick after getting the vaccine then the government should pay for their daily wages. They should not be losing out on their day’s meal just because they've gotten the vaccine. There has to be some provision for people to take the vaccine and then if they are not well then they should be taken care of." (Participant #9)

Overall, results from the survey and interviews suggested the use of multi-pronged strategies for targeted public health messaging to promote COVID-19 vaccine uptake. Inclusive educational tools, especially for the most vulnerable and marginalized, need to be employed. Participants suggested that targeting these groups of people is critical to combating misinformation and ignorance regarding the pandemic to support their well-being. Additionally, the specific focus needs to be given to economically weaker areas to instil trust in the COVID-19 vaccines and promote their uptake.

## Discussion

The goal of this study was to identify the factors that affect COVID-19 vaccine hesitancy, identify barriers that affect vaccination attitudes and understand how to improve vaccine uptake. Overall, more participants displayed pro-vaccine attitudes than anti-vaccine attitudes. The main reasons for expressing vaccine hesitancy included uncertainty and concerns regarding the effectiveness of the vaccine, fear of side effects, unclear and insufficient information about the vaccines, insufficient financial support post-vaccination for daily wage workers, altered risk perception and the reliance on home remedies for protection against the virus. In contrast to the western counties, a higher level of public trust and confidence in the Modi government in this participant sample certainly helped India in its fight against the COVID-19 pandemic [24]. While a study conducted in the United Kingdom suggested that people were concerned about the ingredients of the COVID-19 vaccines [25], this study with older adults shows that these concerns were not important in the Indian context.

The tide of vaccine-related misinformation is a critical barrier that needs to be addressed to increase vaccine uptake in India, especially for middle-aged and older adults [4]. False and misleading claims about the COVID-19 vaccine being dangerous and harmful have cast doubt, preventing people from getting vaccinated [4]. During the interviews, several rumours about vaccines leading to infertility, blood clots, disruption of menstrual cycles, the risk for pregnant women, and the fear of death emerged as some of the key reasons for vaccine refusal and vaccine-hesitant attitudes. Speculations about side effects combined with perceived lower severity of the virus were shown to reduce trust in the COVID-19 vaccines. Notably, the primary source of information for the majority of the participants was social media platforms like WhatsApp, Facebook, Instagram, and Twitter. This is important, as the participants in this study were older adults who are often assumed to have low literacy levels when it comes to digital technology. Studies reveal that people who rely on social media for news are more likely to have misconceptions about the COVID-19 vaccine [26]. This study confirms this finding, but also shows that older adults in India might also be exposed to messages on social media and be influenced by misinformation. 

Among the interview participants, three out of four people who displayed vaccine-hesitant attitudes lived in rural communities. Concerns about infertility, blood clots, breast cancer, heart attacks and the reliance on Ayurveda and homeopathic treatment were common among this group. There is a rising inequity for COVID-19 vaccinations between urban and rural India with unequal access to vaccines, along with the differences in vaccine procurement and allocation policy between the state and central governments [27]. The “liberalized” vaccine policy has enabled people with access to the Internet to book vaccination slots but excluded those without access from getting vaccinated. This is an important consideration for middle-aged and older adults who may not be technologically literate to access online services. This study shows that in addition to the challenges with physical access to the vaccine, rural residents might also have less access to reliable information about the vaccine, a challenge that can be addressed only with an increase in educational campaigns targeting this specific demographic group.

An important finding of this study was that greater vaccine-hesitant attitudes were found in men when compared to women. This is not consistent with findings in the literature where women were found to be more vaccine-hesitant than men [28-29]. However, this can be attributed to the findings from other studies that reported that women were more adherent to medical recommendations and are more cautious about their health [30]. This positive trend is likely to benefit the Indian population as women are more commonly the caretakers at home and having pro-vaccine attitudes can potentially benefit their families. However, given that the participants in this study had higher educational qualifications and greater awareness about health when compared to the overall population in India, this finding should be interpreted with caution.

The perceptions about COVID-19 in India have changed since the second wave of the pandemic. In 2021, the Delta variant led to millions of hospitalizations and deaths and left several people without medical treatment [4]. The Omicron variant, which became the predominant stain afterwards, was relatively mild and thus caused fewer hospitalizations and deaths. The interviews for this study were conducted during the third wave of the COVID-19 pandemic when a large number of adults were already double-vaccinated and some had even received their third shot of the vaccine. Findings from the study show that although there were some individuals who challenged the efficacy of COVID-19 vaccines, the majority still agreed that they were effective enough to reduce complications post-infection.

The participants also provided interesting insights on increasing COVID-19 vaccine uptake. Door-to-door and mass information campaigns were offered as useful strategies to promote vaccines and stop the spread of misinformation. Specifically, the ‘HarGharDastak’ campaign for door-to-door vaccination needs to become more widespread, and, according to the participants, might have the most positive impact in rural areas. It would also help to offer some targeted messaging regarding COVID-19 vaccines’ misinformation, such as the fears of disruption of menstrual cycles and breast cancer in women and infertility in men, by relying on the voices of celebrities and famous personalities that evoke a high level of trust in population. Moreover, there is a need to establish support for daily-wage earners and migrant workers who might be reluctant to get a vaccine because of a temporary inability to work post-vaccination. Holding support camps for these groups is essential for maximum vaccination coverage.

This study has several limitations. First, given the pilot nature of this study and the use of a small, convenience sample, the findings presented here cannot be generalized to the larger population of India. Another limitation embedded in the research design was the timing of data collection. For logistic reasons, the survey was conducted during the time India struggled with a more deadly Delta variant, whereas the interviews were conducted during the spread of the Omicron variant. The timing of the survey and the interview could have impacted the responses provided by the participants and their attitudes towards vaccinations. Finally, since the survey was offered on the Whatsapp platform, it was not accessible to those individuals who may have had limited access to the internet. Notwithstanding these limitations, this study offered some interesting insights about the views on COVID-19 among middle-aged and older adults in India, which can be utilized to develop a larger-scale studies to learn more about vaccine attitudes in this demographic.

Overall, this exploratory pilot study identified key drivers for vaccine acceptance, which included the perceived health benefits of the COVID-19 vaccine, offering it free of charge, maintaining trust in the government and health care system, and facilitating access to reliable information regarding the COVID-19 vaccination. This study also offered some insights on how to address the gap in the knowledge about COVID-19 vaccines among older adults in India, which, hopefully, can impact the decision-making of their families.

## Conclusions

COVID-19 vaccines are an important public health intervention in the fight against the pandemic. The findings of this study suggest that the majority of older adults who took part in this pilot study are vaccinated against COVID-19 and/or have positive attitudes towards COVID-19 vaccines. However, vaccine hesitancy is still a concern, especially among those residing in rural and remote areas. It is important to note that the contributions from this paper are only applicable to the sample in this study and more research is required to understand the diverse perceptions of middle-aged and older adults in India. Findings from this sample showed that improved health communication, including non-digitalized information about COVID-19 vaccines, can improve the uptake of vaccines. Focusing on middle-aged and older adults is critical for ensuring that the health promotion messages are an effective way of reaching out to families and reducing vaccine-hesitancy attitudes among this population.
